# Determinants of periodontal health in pregnant women and association with infants’ anthropometric status: a prospective cohort study from Eastern Uganda

**DOI:** 10.1186/1471-2393-12-90

**Published:** 2012-09-05

**Authors:** Margaret Wandera, Anne N Åstrøm, Isaac Okullo, James K Tumwine

**Affiliations:** 1 Department of Dentistry, Makerere University, Makerere, Uganda; 2 Department of Clinical Dentistry, Faculty of Medicine and Dentistry, University of Bergen, Bergen, Norway; 3 Center for International Health, University of Bergen, Bergen, Norway

**Keywords:** Pregnancy, Oral health, Child, Anthropometric status

## Abstract

**Background:**

Preterm-low birth weight delivery is a major cause of infant morbidity and mortality in sub Saharan Africa and has been linked to poor periodontal health during pregnancy. This study investigated predisposing and enabling factors as determinants of oral health indicators in pregnancy as well as the association between periodontal problems at 7 months gestational age and the infants’ anthropometric status.

**Method:**

A community –based prospective cohort study was conducted in Mbale, Eastern Uganda between 2006 and 2008. Upon recruitment, 713 pregnant women completed interviews and a full mouth oral clinical examination using the CPITN (Community Periodontal Index of Treatment Need) and OHI-S (Simplified Oral Hygiene) indices. A total of 593 women were followed up with anthropometric assessments of their infants 3 weeks after delivery. Multiple logistic regression analyses were used to identify independent determinants of periodontal problems and use of dental services during pregnancy. Analysis of covariance (ANCOVA) was used to investigate the relationship between periodontal problems and the child’s anthropometric status in terms of wasting, underweight and stunting.

**Results:**

A total of 67.0% women presented with periodontal problems, 12.1% with poor oral hygiene, 29.8% with recent dental visit and 65.0% with periodontal symptoms. Of the infants, 2.0% were wasted, 6.9% were underweight and 10.0% were stunted. The odds ratio of having CPI > 0 increased with increased maternal age and single marital status, and was lower in primiparous women and those who used mosquito bed nets. Mean wasting scores discriminated between mothers with CPI = 0 and CPI > 0 as well as between mothers with good and poor OHI-S scores.

**Conclusions:**

Socio-demographic factors and information about oral health were associated with oral health indicators in pregnant women. Second, the height- for- age status at 3 weeks postpartum was worse in infants of mothers having periodontal problems and poor oral hygiene during pregnancy. Efforts to prevent oral diseases during pregnancy should be part of the local state and national health policy agenda in Uganda.

## Background

One in four adults suffers from periodontal disease and pregnancy is associated with increased susceptibility [1-5]. Pregnancy affects a woman’s hormonal balance and acts as a modifying factor of the pathogenesis of periodontal disease [6]. Although pregnancy does not cause periodontal diseases, numerous studies have confirmed increased gingival inflammation occurring between the second and eight months of gestation [6]. Thus, pregnant women have a higher incidence of gingivitis compared with their non-pregnant counterparts and the prevalence rates vary between 36% and 100% [7,8]. A recent study of pregnant women in Brazil revealed a prevalence of periodontal disease of 47% indicating a strong need for initiating oral care during early pregnancy [2]. Lieff et al. [9], observed an increase in attachment loss between the moment women enrolled into their study at <26 weeks gestation and at delivery. Similar results have been reported from other studies conducted in developed as well as developing countries [10-17]. Nevertheless, a number of case-control studies reported no difference in periodontal health between pregnant subjects and non- pregnant controls, leaving findings in this area inconsistent and conflicting.

Apart from the effects of hormonal changes, other factors such as HIV infection, lack of dental care, poor oral hygiene, smoking, low educational level, low employment status, increased age and ethnicity contribute to a worsened periodontal condition during pregnancy [2,18]. Lieff et al. [9] observed that black women were more likely than white women to have periodontal disease both at enrollment into their study and delivery. Taani et al. [11] reported more aggravated at periodontal condition in pregnant women compared with non-pregnant controls even after adjusting for the effect of increasing age, long standing poor personal hygiene and changed psychological status. Unemployment, reflecting low socio-economic status, inaccessibility to dental care and unawareness of oral hygiene was significantly associated with increased gingival inflammation scores. There is a lack of research on socio-behavioral and clinical determinants of pregnant women’s periodontal status and oral hygiene, particularly in developing countries.

Recent meta-analytical reviews have reported a positive association between maternal periodontitis and adverse pregnancy outcomes, such as prematurity, low birth weight infants and preeclampsia [19-22]. Both case control- and prospective studies have shown that periodontitis precedes preterm birth [23,24]. Severe periodontal disease in pregnant mothers has also been reported to be linked with low birth weight at full term delivery in low-and middle income populations [23,24]. A recent survey of pregnant women in Tanzania did not confirm the hypothesis of periodontal disease as a risk factor for preterm low birth weight [17]. Studies on treatment of periodontal disease during pregnancy are conflicting in that some have shown a reduction in the rate of preterm births, whereas others have not [24]. Low birth weight infant delivery, defined by the World Health Organization as a birth weight of <2500 gram presents a major health problem in developing countries. Low-birth weight babies are at increased risk of serious health problems, lasting disabilities and even death. Preterm-low birth weight is the second leading cause of infant death and its incidence continues to rise [5,25,26]. In the Ugandan population, 24% of neonatal deaths are purported to be due to preterm births [27].

### Purpose

Information on periodontal status, oral hygiene and use of dental care during pregnancy emanating from sub- Saharan Africa is rare. Few studies have investigated the relationship between oral health indicators in pregnant mothers and birth outcomes in this socio-cultural context. The present study set out to describe clinical- and self-reported aspects of periodontal- and oral hygiene status as well as use of dental care services in relation to predisposing (i.e. place of residence, age, education, marital status, parity and previous still births) and enabling factors (i.e. antenatal care, information received about teeth) among pregnant women in an ethnically homogenous black African population. The relationship between oral health indicators at 7 months of gestational age and anthropometric status of infants 3 weeks post- partum was also examined.

## Methods

Participating women were members of a multi-center cluster-randomized behavioral intervention trial: Safety and Efficacy of Exclusive Breast feeding Promotion in the era of HIV in Sub Saharan Africa –PROMISE EBF (Id NCT00397150 at http://clinicaltrials.gov) conducted in Uganda, Burkina Faso, Zambia and South Africa. The aim of PROMISE EBF was to develop and test an intervention to promote exclusive breastfeeding, to assess its impact on infants in African contexts and to strengthen the evidence base regarding optimal duration of exclusive breastfeeding [28]. In Uganda, the Mbale district was purposively selected as the intervention site. The units for randomization were clusters made up of 1–2 villages with an average of 1000 inhabitants (35 infants per year given a birth rate of 3.5%). All pregnant women in 24 clusters (18 rural and 6 urban), were eligible for the study. Clusters were selected according to accessibility in terms of being on a main road with reasonable standard especially during the rainy season, access to church, school, trading center and water from the village well. The women were recruited into the PROMISE EBF study between January 2006 and June 2008. There were a total of 6 interviews and 1 oral examination scheduled for each participant: a recruitment interview and a clinical oral examination at 7 months of gestational age, followed by interviews of mothers and anthropometric measurement of infants at 3-, 6-, 12-, and 24 weeks post- partum. Women who did not intend to breastfeed and infants born with serious diseases or deformities that prevented breastfeeding were excluded from participation.

A total of 886 pregnant women were eligible for the study and information was obtained from 877 of the participants (participation rate 98.9%) (mean age 25.6, SD 6.4). Of these, 713 women participated in the oral interview and clinical oral examination conducted between 2006 and 2008. The number of participants satisfied a sample size of 800 pregnant women calculated for the oral sub study, assuming a prevalence of tooth loss (i.e. at least one tooth lost) of 50%, a precision of 0.05 and a design effect of 2. At three weeks post- partum, 635 mothers were re-interviewed in their homes and 593 of their infants were assessed for anthropometric status. The procedures of recruitment and participation are detailed in the PROMISE EBF study profile [18,29-31]. Ethical Clearance was obtained from the Ethical Review Board, Faculty of Medicine, Makerere University. Written consent was obtained from all participants in the study and prior to each examination and interview a verbal consent was obtained.

### Interviews

Structured interviews, designed on a desk- top PC using Epi-Handy and then down loaded to handheld computers, were conducted in face- to- face settings with participating women at household level. The interview schedules were developed in English and translated into the local language of Lumasaba. Oral health professionals reviewed the interview schedule for semantic, experiential and conceptual equivalence. Sensitivity to culture was considered and words selected appropriately. The interview schedules were piloted before administration. Gilbert [32] has described a model that explicitly conceptualizes the relationship of oral health conditions with predisposing and enabling immutable and mutable factors, such as socio-demographics, parity (predisposing factors) and exposure to oral health counseling during pregnancy (enabling factors). This model was used to guide the identification of exploratory variables utilized in this study. The following self-reported outcome variables were assessed: Use of dental care was assessed by asking “When was your last dental visit “and recorded as (0) never attended and (1) within the recent 6 months or more seldom. Periodontal symptoms were measured by five items in terms of “During the previous 3 months have you experienced: bleeding gums when brushing/eating, spontaneous bleeding from gums, pain in gums, changed gum color, swollen gums?” Each item was recorded as (0) no and (1) yes. A sum score was constructed by adding the items and dichotomizing based on a median split into (0) mild symptoms, (1) severe symptoms. Predisposing explanatory variables were identified as place of residence, age, educational level, marital status, parity and previous birth outcome experience. Enabling explanatory variables related to information received about own teeth/child’s teeth were assessed by 6 items in terms of “have you ever received information regarding how to take care of own teeth from health worker, dentist, radio, MCH clinic, newspapers and other sources?”. Each item was recorded as (0) no and (1) yes. A sum score was constructed and dichotomized into (0) never received information and (1) received information from one or more sources. A similar score was computed for information received about children’s teeth. Attendance at antenatal care and use of bed nets were each recorded as (0) no and (1) yes. The predictor variables employed and their coding are depicted in Table 1.

**Table 1:** Frequency distribution of self-reported and clinically assessed exploratory variables at 3 weeks post- partum (total n = 877)

***Variable***	***%*** ***(n)***
*Mothers’ variables*	
Residence	
Urban	26.7 (231)
Rural	73.3 (633)
Age	
<20 yrs	26.3 (22)
21-30 yrs	53.9 (454)
31-45 yrs	19.8 (167)
Educational level	
Primary school	67.7 (531)
Secondary and above	32.3 (253)
Marital status:	
Single	8.2 (70)
Married	61.8 (528)
Cohabiting	30.0 (256)
Parity	
One or more births	77.3 (660)
Primiparous	22.7 (194)
Attended antenatal care	
No	26.7 (227)
Yes	73.3 (617)
Use a bed net	
No	50.2 (423)
Yes	49.8 (419)
Received Information on own teeth	
No	73.9 (589)
Yes	26.1 (208)
Received Information babies teeth	
No	80.6 (624)
Yes	19.4 (155)
CPI ( total n=713)	
= 0	33.0 (235)
≥1	67.0 (478)
OHI-S	
Good	87.9 (624)
fair/poor	12.1 (86)
Dental attendance	
Never	70.2 (566)
within the last 6 months	29.8 (240)
or more seldom	
Periodontal symptoms	
fair	34.6 (274)
severe	65.4 (519)
*Infant anthropometric variables* (total n=519)	
Weight for height <-2	2.0 (11)
Weight for age <-2	6.9 (41)
Birth weight <2.5kg	9.0 (19)
Height for age <-2	10.0 (59)

The total number of the various categories does not add to 877 due to missing values.

### Clinical oral examination

A trained and calibrated dentist (MW) carried out oral examinations under field conditions based on the WHO criteria [16]. An assistant recorded the data on a prepared record sheet. All fully erupted permanent teeth were scored excluding third molars. All examinations were performed at household level with subjects seated on the left hand side of the examiner who used a headlamp as source of illumination. No radiographic examination or drying of teeth was performed. The periodontal status was assessed using a plane –faced dental mirror and a specially designed lightweight CPITN probe with a 0,5 mm ball tip. Using the epidemiological part of the CPITN, the Community Periodontal Index (CPI) [15] with 10 index teeth (17,16,11,26,27,47,46,31,36 and 37) and 6 sextants per individual, four indicators of periodontal status were applied. Periodontal pockets were measured from the edge of the free gingiva to the bottom of the pocket. The criteria used were; healthy (code 0), bleeding on probing observed (code 1), calculus detected during probing (code 2), pocket 4–5 mm (code 3) and pocket >5 mm (code 4). Each index tooth was scored on two sites (buccal and lingual) and each sextant was scored according to its highest CPI score. In accordance with the hierarchical assumption, teeth with score 3 were assumed positive with respect to bleeding and calculus whereas teeth with score 2 were assumed positive with respect to bleeding [33]. Prevalence of healthy-, bleeding-, calculus and pocket sextants was assessed as the percentage of subjects affected (having at least one sextant affected). Severity of periodontal condition was assessed by the mean number of sextants having CPI code 0–3 and by the mean number of sextants in affected persons. The CPI recordings, total CPI, were also presented as the percentage distribution of dentate subjects according to the highest score in the mouth. *Oral hygiene status* was measured using the Oral Hygiene Index- Simplified (OHI-S) by Greene and Vermillion [34]. The index has two components (debris index- simplified (DI-S) and calculus index – simplified (CI-S). Debris and calculus were graded on a numeric scale from 0 to 3, divided by number of sites recorded and categorized in terms of low debris/calculus (0) (score 0.0-0.67) and fair debris/calculus score (1) (score 0.69-1.67).

### Anthropometric status

A total of 593 infants were examined regarding their weight and recumbent length in accordance with the WHO recommendations [35]. Standardized 25 kg portable Salter Spring scales measuring to the nearest 0.1 kg were used to determine weight. Recumbent length was measured to the nearest 0.1 cm with specially designed length boards. Using the WHO Child Growth Standards [35], anthropometric indices were constructed on the basis of weight, length, age and sex. Wasting was defined as weight-for-height z-scores (WHZ) < −2 SD, stunting as height-for-age z-scores (HAZ) < −2 SD and underweight as weight-for-age z-scores (WAZ) < −2 SD [35]. Predisposing and enabling variables used as explanatory variables in the analyses as well as the outcome variables and the numbers of subjects (%) according to categories are depicted in Table 1.

### Reproducibility

Duplicate clinical examinations were carried out with 50 mothers considered to be representative of the study participants after a period of one month. Kappa values for indicators of periodontal condition ranged from 0.48 (CPI index tooth 11) to 0.85 (CPI index tooth 31). The figures indicate moderate to good intra examiner reliability [36].

### Statistical analysis

Data was entered into the Epi-Handy program on the handheld computers and analyzed using SPSS version 19.0 (Chicago, IL, USA). Cross tabulation, chi square statistics and ANOVA were used to assess bivariate relationships. Multiple variable logistic regression analyses were conducted with maternal oral health indicators as dependent variables using the logit model and 95% confidence intervals (CI) for the odds ratios. ANCOVA was used to assess the relationship between children’s anthropometric status and mothers’ periodontal status during pregnancy.

## Results

### Description of the study sample

A total of 877 (mean age 25 yr, SD 6.4) pregnant women consented to participate in the recruitment interview of whom 713 (mean age 25 yr, SD 6.0) underwent oral clinical examination, and 635 completed post- partum interview 3 weeks after delivery. As depicted in Table 1, 26.7% of the pregnant women were resident in urban areas whereas 73.3% in rural, 32.3% reported education above primary school level, 61.8% were married and 73.3% had visited antenatal care during pregnancy.

### Non- response analyses

Of the 877 women who completed the recruitment interview, 164 (18.7%) did not undergo clinical oral examination. To analyze the possibility of selection bias, a comparison was made of the socio-demographic characteristics of participants (n = 713) and non-participants (n = 164) of the oral clinical examination. The frequency distributions of age, education, and parity were similar between the two groups. However, 78% of non-respondents versus 68% of the respondents had never visited a dentist (p < 0.05). Socio-demographic characteristics were also compared between participants (n = 635) and non- participants (n = 242) of the 3 week post- partum interview. No substantial differences were found except that there was a higher percentage of urban residents among non-participants than among participants (34% versus 24%, p < 0.05).

### Maternal oral health indicators and infants’ anthropometric status

A total of 67% mothers presented with CPI score ≥ 1, 12.1% had fair to poor OHI-S scores, 28.8% had received dental attendance within the previous 6 months and reported severe periodontal symptoms (Table 1). The mean number of sextants with CPI score 0, CPI score 1 or higher and CPI score 2 or higher amounted to 4.5 (95% CI 4.4-4.6), 1.4 (95% CI 1.2-1.5) and 1.2 (95% CI 1.2-1.3), respectively (not in table). The proportion of women in urban areas having a total CPI scores of 0 was 37.0%, 4,4% had a CPI score of 1, 56.9% had a CPI score of 2 and 1.7% had a CPI score of 3. In rural areas these percentages were 31.7%, 2.8%, 65.3% and 0.2% respectively (not in table). Compared to participants in the youngest age group, higher proportions of older women showed CPI ≥ 1, reported severe periodontal symptoms, had recently had a dental visit and presented with poor OHI-S scores. Compared to their primiparous counterparts, larger proportions of multiparous women presented with CPI ≥ 1 and periodontal symptoms (Table 2). Of the infants, 2.0% showed WHZ < −2 (wasting), 6.9% showed WAZ < −2 (underweight) and 10.0% showed HAZ < −2 (stunting), respectively. With few exceptions, anthropometric measures did not vary systematically with predisposing and enabling factors (Table 3).

**Table 2:** Maternal oral health indicators by predisposing and enabling factors

**Predisposing factors**		**CPI≥1**	**Periodontal**	**Dental visiting**	**OHI-S-poor**
		**% (n)**	**symptoms**	**<6 months**	**% (n)**
			**% (n)**	**% (n)**	
Place of residence:	Urban	63.0 (14)	60.2 (121)	37.3 (76)	10.0 (18)
	Rural	68.3 (360)	67.2 (393)	27.2 (162)**	12.8 (67)
Age:	≤ 20 yr	52.0 (92)	53.9 (110)	21.8 (45)	7.4 (13)
	21-30 yr	68.4 (253)	68.6 (284)	30.1 (127)	10.3 (38)
	31-45 yr	83.0 8122)**	70.5 (110)**	37.7 (60)*	23.3 (34)**
Education:	Primary	66.9 (291)	66.1 (143)	27.3 (136)	12.0 (52)
	Secondary and above	64.7 (141)	61.6 (143)	36.3 (85)*	9.7 (21)
Marital status:	Single	77.8 (42)	57.1 (32)	23.2 (13)	11.1 (6)
	Married	69.8 (312)	66.4 (322)	29.4 (146)	13.9 (62)
	Cohabiting	58.1 (118)**	64.8 (158)	30.9 (76)	8.5 (179
Parity:	One child or more	70.9 (389)	68.3 (411)	33.1 (203)	13.6 (74)
	Primiparous	53.5 (83)**	55.2 (101)**	17.3 (32)**	7.1 (11)*
Previous still birth:	No	67.4 (246)	69.3 (278)	34.2 (140)	12.7 (46)
	Yes	78.1 (139)*	66.5 (129)	31.0 (61)	15.8 (28)
**Enabling factors**					
Attended antenatal care:	No	70.8 (126)	64.3 (128)	23.6 (48)	12.9 (23)
	Yes	65.8 (340)	65.6 (378)	31.6 (186)*	12.1 (62)
Use of bed net:	No	72.1 (248)	66.7 (256)	25.2 (99)	15.2 (52)
	Yes	62.1 (218)**	63.9 (250)	33.9 (134)*	9.5 (33)*
Received Information on own teeth:	No	67.1 (324)	62.7 (360)	25.6 (150)	12.1 (58)
	Yes	68.1 (124)	72.5 (150)*	39.9 (83)**	14.4 (26)
Received Information on child’s teeth:	No	68.3 (366)	63.0 (395)	27.9 (178)	
	Yes	63.6 (82)	74.7 (115)**	35.5 (55)*	

* p < 0.05, ** p < 0.01.

**Table 3:** Anthropometric status at 3 weeks post- partum by predisposing and enabling factors in mothers’ during pregnancy at 7 months of gestational age (n = 593)

		**WHZ>-2**	**WAZ<-2**	**HAZ<-2**	**Birth weight**
**Predisposing/enabling**		**% (n)**	**% (n)**	**% (n)**	**<2500gram**
					**% (n)**
Place of residence:	Urban	2.2 (3)	9.4 (13)	10.0 (15)	8.0 (7)
	Rural	1.9 (8)	6.0 (27)	9.8 (44)	8.1 (10)
Age:	≤ 20 yr	4.2 (6)	9.2 (14)	8.5 (13)	7.4 (4)
	21-30 yr	1.7 (5)	5.9 (18)	9.6 (29)	9.5 (11)
	31-45 yr	0.0 (0)*	6.6 (8)	12.2 (159	7.7 (3)
Education:	Low	2.6(9)	6.5 (24)	8.4 (31)	10.4 (11)
	High	1.3 (2)	8.6 (15)	12.6 (22)	7.4 (79
Parity:	One child or more	2.1 (9)	5.7 (26)	9.6 (44)	8.3 (12)
	None	1.6 (2)	10.8 (14)*	9.9 (139	9.4 (6)
Attended antenatal care:	No	5.7 (8)	5.3 (8)	8.7 (13)	7.3 (3)
	Yes	0.7 (3)**	7.4 (32)	10.2 (44)	9.1 (15)
Received Information about own teeth:	No	2.3 (9)	5.7 (23)	8.4 (34)	7.7 (11)
	Yes	1.4 (2)	10.8 (16)*	12.8 (19)	12.9 (8)

* p < 0.05, ** p < 0.01.

### Multivariable analyses

Adjusted OR (95% CI) from logistic regression analyses is presented in Table 4. All predisposing and enabling factors that showed a statistically significant association with maternal oral health indicators in bivariate analyses were included in the multivariable models.Tthe ORs for having CPI ≥1 were higher for women aged 21–30 yrs (OR = 1.8, 95% CI 1.2-2.8), and 31–45 yrs (OR = 3.7, 95% CI 2.1-6.6) compared with their counterparts below 21 yrs. The ORs for having CPI ≥ 1 were lower in married women (OR = 0.4 , 95% CI 0.2-0.9) and cohabiting women (OR = 0.3, 95% CI 0.1-0.8) compared to single mothers. Primiparous mothers and mothers using mosquito bed nets were less likely to have CPI ≥ 1 than their counterparts in the opposite groups. Older women (OR = 1.7, 95% CI 1.0-2.8)) and women who had received oral health information during pregnancy (OR = 1.5, 95% CI 1.0-2.5) were more likely than their counterparts to report periodontal symptoms. The ORs for having visited a dentist within the previous 6 months increased with increasing age, higher educational level (OR = 1.6, 95% CI 1.2-2.3), and with oral health information received (OR = 1.7, 95% CI 1.2-2.6). The OR for having visited a dentist was lower for primiparous compared witho multiparous women (OR = 0.4, 95% CI 0.2-0.6).

**Table 4:** Adjusted OR and 95% Confidence intervals (95% CI) from multiple variable logistic regression analyses of maternal oral health indicators by predisposing and enabling factors

		**CPI>0**	**Periodontal Symptoms**	**OHI-S**	**Dental attendance**
		Adjusted	Adjusted	Adjusted	Adjusted
**Predisposing**		OR (95% CI)	OR (95% CI)	OR (95% CI)	OR (95% CI)
					
Residence	Urban				
	Rural	-	-		1
		-	-		0.6 (0.4-1.0)
Age	<20 yr	1	1	1	1
	21-30 yr	1.8 (1.2-2.8)	1.6 (1.1-2.4)	1.3 (0.6-2.6)	1.0 (0.7-1.7)
	31-45 yr	3.7 (2.1-6.6)	1.7 (1.0-2.8)	3.2 (1.5-6.8)	2.1 (1.2-3.5)
Education	Low				1
	High				1.6 (1.2-2.3)
Marital status	Single	1			
	Married	0.4 (0.2-0.9)			
	Cohabiting	0.3 (0.1-0.8)			
Parity	multi	1	1	1	1
	none	0.6 (0.3-0.9)	0.7 (0.5-1.1)	0.6 (0.3-1.3)	0.4 (0.2-0.6)
**Enabling factors**					
					
Attended antenatal care:	No				1
	Yes				1.2 (0.8-1.7)
Use of bed net:	No	1		1	1
	Yes	0.6 (0.4-0.9)		0.5(0.3-0.9)	1.3 (0.8-1.9)
Information own teeth:	No		1		1
	Yes		1.2 (0.8-1.9)		1.7 (1.2-2.6)
Information baby teeth:	No		1		1
	Yes		1.5 (1.0-2.5)		0.9 (0.6-1.6)

As depicted in Table 5, adjusting for maternal age revealed average wasting (HAZ) scores that discriminated statistically significantly between mothers with (−0.26, SD 1.2) and without (−0.48, SD 1.2) CPI ≥ 1 and between mothers having good ( −0.36 SD 1.2) and poor/fair OHI_S scores (−0.70 SD 1.1) (p < 0.05) at 7 months of gestational age.

**Table 5:** Anthropometric status at 3 weeks post- partum by maternal oral health indicators recorded at 7 months of gestational age

	**HAZ**	**WAZ**	**WHZ**
CPI=0	-0.26 (1.2)	-0.32 (1.1)	-0.31 (1.2)
CPI≥1	-0.48 (1.2)*	-0.33 (1.1)	-0.05 (1.3)*
OHI-S<1.2 (low)	-0.36 (1.2)	-0.31 (1.1)	-0.15 (1.2)
OHI-S ≥ 1.2 (high)	-0.70 (1.1)*	-0.45( 1.2)	-0.03 (1.3)

Values are mean (SD).n = 519 * p < 0.05.

## Discussion

Periodontal disease during pregnancy was influenced by greater maternal age, single marital status, being primiparous, having received oral health information and using mosquito bed nets. Wasting status at 3 weeks postpartum was worse in infants of mothers with periodontal problems. Studies of periodontal condition during pregnancy vary with respect to ethnicity and socio-demographic features of the populations investigated. Definitions of disease parameters, such as gingival bleeding, probing pocket depths and attachment and alveolar bone loss and the number of sites per tooth and number of teeth examined per individual also vary across studies [2,5,9]. These inconsistencies influence the results and limits valid comparisons between studies [37]. Numerous studies have shown that estimates of prevalence and severity as well as distributional characteristics of periodontal condition vary according to the method used for recording [37]. In this study, the CPI, [15,16] was deemed an appropriate screening system since the clinical examinations were carried out at household settings. Although CPI scores 1 and 2 primarily reflect gingival inflammation, bleeding and calculus, conditions that do not necessarily progress to periodontal destruction, this index has been used extensively to reflect periodontal health in various populations in Europe, Kenya, Tanzania and Ethiopia [3,4].

The overall findings of this study, characterized by low prevalence of bleeding, high prevalence of calculus and infrequent occurrence of shallow pockets, is consistent with findings in African populations in general [3,4]. Studies using the CPITN have indicated that the prevalence of severe periodontal disease is low in the general populations of sub Saharan Africa [3,4,13,14]. Moreover, the oral hygiene has been described as poor with plaque and calculus accumulating with increasing age [3]. The low prevalence of periodontal condition revealed by the present study was supported by a moderate prevalence of women showing poor oral hygiene status (12.1%), but was at odds with the substantial rate of self- reported periodontal symptoms (65.4%). Considering the limitations associated with the CPI scoring system and that this method does not provide a complete measure of periodontal disease, the present study demonstrated shallow pockets of 4–5 mm amounting to 1.7% in the urban and 0.2% in the rural participants, whereas 67% presented with any sign of periodontal disease (CPI ≥ 1). Miyazaki et al. [7] found a prevalence of 31% shallow pockets in pregnant Japanese women, whereas the prevalence of any periodontal disease amounted to 97%. Compared to an earlier study of Ghanaian pregnant women with mean number of bleeding sextants amounting to 3.2 and 1.9 in second and third trimester [14], the corresponding figure of 1.2 observed in this study was moderate. Consistent with the present results, other studies from sub- Saharan Africa have reported on shallow pockets among postpartum women in all age groups, whereas in pregnant women shallow pockets have been observed in 25-45-year-olds during the second trimester and in 35–45 year -olds during the third trimester [17]. Some studies have documented no worsening of clinical attachment loss during pregnancy, but a progressive increase in plaque and bleeding scores with a peak level occurring at seven months of gestational ages [10-12]. Since only one clinical examination was performed, it was not possible to affirm any exacerbation of pre-existing or early periodontal disease among the pregnant women investigated in the present study. Moreover, using the number of women with bleeding, calculus and pockets instead of attachment loss as periodontal outcome variables focuses the extent of the infection at the time of the survey more than the consequences of previous disease processes.

Several determinants of maternal oral health indicators were identified, suggesting that subgroups of women may be at increased risk of deteriorating oral health during pregnancy. A pattern of positive associations between periodontal disease and age has been found in numerous studies globally and among Ugandan students where similar age distributions were observed regarding both aggressive- as well as chronic periodontitis [38,39]. Moreover, the present findings accord with a strong social gradient in periodontal disease reported previously [2,18]. In this study, use of mosquito bed nets and marital status were used as surrogates of socio-economic status [40]. Previous studies with the cohort of Mbale women revealed that perinatal deaths were associated with preterm birth, malaria and complicated deliveries [27]. In this study, pregnant women who reported use of mosquito bed nets were less likely to have periodontal disease while single mothers were more likely to have poor OHI-S status. In accordance with some previous investigations but at odds with others, parity was positively associated with periodontal disease. This association was maintained even after adjusting for relevant covariates such as the possible effect of increasing age which is often associated with multiple pregnancies [11,39]. Scheutz and Baelum [13] reported no association between parity and tooth loss, whereas attachment loss was found to be more pronounced in multiparous than in primiparous women in Tanzania. A positive association between parity and periodontal disease might be attributed to accumulated tissue destruction across time rather than an intrinsic parity- related abnormality.

Although about one third of the pregnant women investigated had their last dental visit within the previous 6 months, primiparous women were more likely to document such a visit than their multiparous counterparts. The limited frequency of dental visits corroborates findings pertaining to pregnant women from other cultures [41,42]. One possible explanation is that pregnant women tend to avoid dental care because they believe poor oral health to be a routine consequence of pregnancy and because of fear that dental care would harm the fetus [5]. Although The American Academy of Periodontology recommends maintenance of oral hygiene during pregnancy, only about 20-40% of pregnant US women receive dental care [42]. In this study, higher proportions of women educated above primary school level and having received information on oral health had visited a dentist. Thompson et al. [39] observed that women with a low level education were less likely than their higher educated counterparts to report tooth cleaning during pregnancy. Participants of the present study came from an area with low economic resources and where the access to dental care is poor. In such settings prevention and treatment of oral diseases has not been strongly advocated [43]. Low rates of dental attendance might be attributed to few available dental clinics and a low priority given to this service in the Ugandan socio-cultural context where prevailing levels of communicable diseases, poverty and restructuring of health systems are more important concerns.

Height- for- age scores at 3 weeks postpartum were significantly lower in infants of mothers having CPI scores > 0 and poor oral hygiene during pregnancy. Under the assumption that poor anthropometric status at 3 weeks post- partum may be one possible consequence of preterm low birth weight, the present results may add to the evidence of a positive relationship between periodontal problems and poor pregnancy outcomes [19-24]. However, this finding should be interpreted with caution as many unmeasured factors, such as feeding practice immediately after birth and infectious diseases, might have confounded the observed association in this study. Moreover, it cannot be ruled out that unaccounted factors such as smoking, maternal weight, anemia and other medical problems may have confounded the estimates presented, either positively or negatively. Future studies involving populations with a low risk of severe periodontitis should enroll larger samples. As the present study was based on recruited women in PROMISE-EBF, it was limited by the sampling methodology used. Thus, the results of this study should not be generalized to the whole population of Ugandan women or to the population of pregnant women in Mbale. However, prospective and interventional studies provide the most important evidence related to a possible relationship between maternal oral health status and infants’ post- partum anthropometric status, and the major strength of this present study is its longitudinal design.

## Conclusion

Socio-demographic factors and oral health information were independently associated with periodontal status, oral hygiene and use of dental care in pregnant women in Mbale Eastern Uganda. Second, the height- for- age status at 3 weeks postpartum was significantly worse in infants of mothers having CPI scores > 0 and poor oral hygiene. Efforts to prevent oral diseases during pregnancy should be part of the local state and national health policy agenda and should focus on modifiable barriers to oral health care. When pregnant women have access to dental care and are motivated to use dental care on a regular basis both mother and child may experience benefits.

## Abbreviations

CPI: Community periodontal index; HAZ: Height-for-age z-score; OHI-S: Oral hygiene index simplified; WAZ: Weight-for-age z-score; WHZ: Weight-for-height z-score.

## Competing interests

The authors declare that they have no competing interests.

## Authors’ contributions

MW is the principle investigator who designed the study, collected the data and performed the main statistical analysis and paper writing, ANÅ acted as the supervisor of MW and participated in the statistical analyses and completion of the paper. IO and JT have both provided valuable guidance during design of the study and data collection. All authors have read and approved the final manuscript.

## Authors’ information

MW PhD is a researcher employed at Makarere University, Uganda. ANÅ is a professor of odontology employed at the University of Bergen, Norway. IO is a researcher employed at Makarere, JT is a medical doctor and professor at Makarere University, Uganda.

## Financial support

The study was part of the EU-funded project PROMISE-EBF (contract no INCO-CT 2004-003660, http://www.promiseresearch.org). It was also financially supported by the Norwegian Research Council funded project *Oral health in a global perspective* (project number 156744)

## List of members for the PROMISE-EBF study group

Steering Committee:

Thorkild Tylleskär, Philippe Van de Perre, Eva-Charlotte Ekström, Nicolas Meda, James K. Tumwine, Chipepo Kankasa, Debra Jackson.

Participating countries and investigators:

*Norway:* Thorkild Tylleskär, Ingunn MS Engebretsen, Lars Thore Fadnes, Eli Fjeld, Knut Fylkesnes, Jørn Klungsøyr, Anne Nordrehaug-Åstrøm, Øystein Evjen Olsen, Bjarne Robberstad, Halvor Sommerfelt

*France:* Philippe Van de Perre

*Sweden:* Eva-Charlotte Ekström

*Burkina Faso:* Nicolas Meda, Hama Diallo, Thomas Ouedrago, Jeremi Rouamba, Bernadette Traoré Germain Traoré, Emmanuel Zabsonré

*Uganda:* James K. Tumwine, Caleb Bwengye, Charles Karamagi, Victoria Nankabirwa, Jolly Nankunda, Grace Ndeezi, Margaret Wandera

*Zambia:* Chipepo Kankasa, Mary Katepa-Bwalya, Chafye Siuluta, Seter Siziya

*South Africa:* Debra Jackson, Mickey Chopra, Mark Colvin, Tanya Doherty, Ameena E Googa, Lyness Matizirofa, Lungiswa Nkonki, David Sanders, Wanga Zembe.

(Country PI first, others in alphabetical order of surname)

## Pre-publication history

The pre-publication history for this paper can be accessed here:

http://www.biomedcentral.com/1471-2393/12/90/prepub
